# Evidence for Chromatin-Remodeling Complex PBAP-Controlled Maintenance of the *Drosophila* Ovarian Germline Stem Cells

**DOI:** 10.1371/journal.pone.0103473

**Published:** 2014-07-28

**Authors:** Jie He, Tao Xuan, Tianchi Xin, Hongbo An, Jinye Wang, Gengchun Zhao, Mingfa Li

**Affiliations:** 1 School of Life Sciences and Biotechnology, Shanghai Jiao Tong University, Shanghai, P R China; 2 School of Biomedical Engineering, Shanghai Jiao Tong University, Shanghai, P R China; National Cancer Institute, United States of America

## Abstract

In the *Drosophila* oogenesis, germline stem cells (GSCs) continuously self-renew and differentiate into daughter cells for consecutive germline lineage commitment. This developmental process has become an *in vivo* working platform for studying adult stem cell fate regulation. An increasing number of studies have shown that while concerted actions of extrinsic signals from the niche and intrinsic regulatory machineries control GSC self-renewal and germline differentiation, epigenetic regulation is implicated in the process. Here, we report that Brahma (Brm), the ATPase subunit of the *Drosophila* SWI/SNF chromatin-remodeling complexes, is required for maintaining GSC fate. Removal or knockdown of Brm function in either germline or niche cells causes a GSC loss, but does not disrupt normal germline differentiation within the germarium evidenced at the molecular and morphological levels. There are two *Drosophila* SWI/SNF complexes: the Brm-associated protein (BAP) complex and the polybromo-containing BAP (PBAP) complex. More genetic studies reveal that mutations in *polybromo*/*bap180*, rather than gene encoding Osa, the BAP complex-specific subunit, elicit a defect in GSC maintenance reminiscent of the *brm* mutant phenotype. Further genetic interaction test suggests a functional association between *brm* and *polybromo* in controlling GSC self-renewal. Taken together, studies in this paper provide the first demonstration that Brm in the form of the PBAP complex functions in the GSC fate regulation.

## Introduction

*Drosophila* oogenesis begins with the asymmetric division of the germline stem cells (GSCs) at the anterior tip of the germarium in the ovary [1]. This division produces one daughter cell retaining the stem cell identity, and another differentiating progeny called cystoblast (CB). Each CB subsequently proceeds with four incomplete mitotic divisions to consecutively form interconnected 2-cell, 4-cell, 8-cell and 16-cell germline cysts. Within the 16-cell cyst, only one germ cell differentiates as oocyte, whereas the remaining 15 become supportive nurse cells [2]. After encapsulated by a monolayer of epithelial follicle cells, the cyst moves out of the germarium to form an egg chamber [3]. Continuous generation of self-renewing GSCs and their differentiating descendant cells for the cyst development are essential for fertility throughout the female fly's lifetime.

In the oogenesis, GSC cell fate is maintained by both extrinsic signals from the niche and intrinsic regulatory machineries. Cap cells (CpCs) in the niche produce BMP-like signal molecule Dpp for activating BMP signaling pathway in GSCs. Active BMP signaling maintains GSC fate by repressing differentiation via transcriptional silence of the differentiation promoting gene, *bag-of-marbles* (*bam*) [4], [5], [6], [7], [8], [9], [10]. In addition to the BMP/Bam pathway, the Nano/Pumilio complex and miRNA pathway are cell-autonomously required for GSC maintenance [11], [12], [13], [14], [15], [16], [17]. Likewise, regulation of stepwise germline differentiation derived from GSCs within the germarium involves intrinsic and extrinsic mechanisms. In the case of cell autonomous mode, a number of molecular markers such as Sex lethal, Bam, Nanos, A2BP1, Bruno and Orb have been identified as key regulators for specific transitions within the differentiation process [4], [12], [18], [19], [20], [21]. Recently, Escort cells (ECs) physically interacting with differentiating germ cells in the germarium were found to promote germline differentiation in a non-cell autonomous manner through restricting BMP signaling inside the GSC niche [22], [23]. Both GSCs and germline cyst development in the germarium have become *in vivo* working platforms for addressing how adult stem cell fate and stem cell-derived cell lineage commitment are regulated [1].

A growing number of evidences have indicated that GSC fate regulation can also occur at epigenetic level. We and others have identified a number of epigenetic factors involving histone modification or chromatin remodeling as regulators for GSC maintenance and germ cell differentiation [24], [25], . In the present study, we extended the investigation to the *Drosophila* SWI/SNF chromatin-remodeling complex. There exists two subtypes of the SWI/SNF complexes in *Drosophila*: the Brahma (Brm)-associated protein (BAP) complex and the polybromo-containing BAP (PBAP) complex [34], [35]. Genetic studies revealed that Brm, the ATPase subunit of the SWI/SNF complexes, is required intrinsically and extrinsically for maintaining GSCs, but not for germline differentiation within the germarium. Mutations in gene *polybromo/bap180*, rather than *osa*, caused a defect in GSC maintenance reminiscent of the *brm* mutant phenotype. We further showed a genetic interaction of *brm* with *polybromo/bap180* in sustaining the GSC population. Thus, we propose that Brm acts in the form of the PBAP complex to control GSC self-renewal in the *Drosophila* oogenesis.

## Material and Methods

### *Drosophila* stocks and genetics

All *Drosophila* strains were maintained and crossed at 25°C unless otherwise stated. The following fly stocks were used in this study:

Canton S (CS) and *w^1118^* strain was used as wild type.

Mutant alleles and transgene: *brm^T362^* [36], *UAS-brm^K804R^* [37], *bap180^Δ86^* [38] (from Jessica E. Treisman), *brm^2^*, *osa^308^*, *osa^2^*, *hsFlp;FRT80B,ubi-GFP* (Bloomington *Drosophila* Stock Center, BDSC), *yw122;FRT82B,ubi-GFP* (gift from Zhao-hui Wang).

RNAi: *UAS-brm-RNAi-B35211*, *UAS-brm-RNAi-B34520*, *UAS-bap180-RNAi-B32840*, *UAS-bap170-RNAi-B26308* (BDSC), *UAS-bap180-RNAi-V108618* (Vienna *Drosophila* RNAi Center, VDRC). The on-target effects of the above RNAi transgenes were molecularly validated based on the RT-PCR quantative assay (Figure S2)

Gal4/UAS: *nos-Gal4.NGT*, *bab1-Gal4* and *tubP-Gal80^ts^* (BDSC), *c587-Gal4* [39] (gift from Yu Cai)

Mosaic clones were generated by mitotic recombination using FLP/FRT system. To generate GSC or germline cyst clones mutant for *brm* or *bap180*, *hsFLP; FRT80B ubiGFP* was crossed to *FRT80B*, *FRT80B brm^T362^*, *hsFLP; FRT82B ubiGFP* was crossed to *FRT82B*, *FRT82B bap180^Δ86^*. Two-day-old female adult progenies with appropriate genotype (*hsFLP; FRT80B ubiGFP/FRT80B brm^T362^* or *hsFLP; FRT82B ubiGFP/FRT82B bap180^Δ86^*) were heat-shocked at 37°C twice a day on three consecutive days for one hour each time. Meanwhile, the progenies from the cross of *hsFLP; FRT80B ubiGFP* and *FRT80B* or *hsFLP; FRT82B ubiGFP* and *FRT82B* were used as FRT controls respectively. Ovaries were then dissected at day 2, 7,14 and 21 after the last heat-shock treatment for the clonal analysis. The FRT clones were identified by the absence of GFP expression.

RNAi-based knockdown experiments were performed by Gal4/UAS binary system [40]. For the spatial-temporally controlled study, the RNAi transgenic line was crossed to *tubP-Gal80^ts^* and *bab1-Gal4*. The females were raised at 18°C until 2 days after eclosion and then shifted to 29°C for a number of days.

### Antibodies and immunofluorescence

Antibody staining was carried out as described previously [41]. The following primary antibodies were used: guinea pig anti-Brm (1∶1000, from Feng Tie) [42], mouse anti- α -Spec (1∶20, DSHB 3A9(323 or M10-2)), mouse anti-Bam (1∶5, DSHB Fly Bag-of-Marbles), rabbit anti-Nanos (1∶1000, from Akira Nakamura), mouse anti-Sxl (DSHB, M114), guinea pig anti-A2BP1 (from Michael Buszczak) [19], mouse anti-Orb (DSHB, orb 4H8), rabbit anti-Bruno (1∶1000, from Mary A. Lilly) [43], rabbit anti-pMad (1∶2000 gift from E. Laufer) [44], rabbit anti-Vasa (1∶200, Santa Cruz sc-30210). Secondary antibodies conjugated with Alexa Fluor 488, 546 (Invitrogen) were used at 1∶1000 dilutions. DAPI (Invitrogen) was used to visualize nuclei.

Confocal images were captured on Leica TCS SP5 laser confocal microscope.

### GSC and UGC identification and statistical analysis

GSCs were identified by the presence of a spectrosome anchored to the CpC contact site. The spectrosome-containing single germ cells in the germarium which are located away from terminal filaments and cap cells were classified as UGCs.

Student's t test and Mann–Whitney test were chosen to calculate p-values.

## Results and Discussion

### The polybromo-containing BAP (PBAP) complex is required intrinsically for GSC maintenance in the *Drosophila* ovary

**Figure 1:**
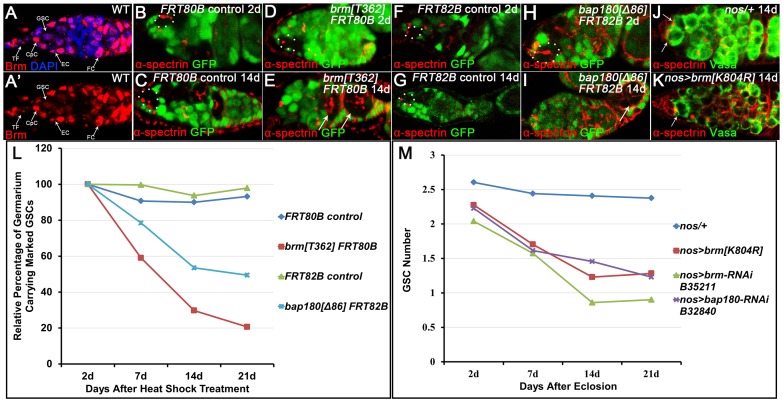
Mutation or reduced expression of *brm* or *polybromo/bap180* in the germline causes a defective GSC maintenance. (A, A′) In the wild type germarium, *brm* is ubiquitously expressed in almost all cell types, predominantly in TFs, CpCs, ECs and follicle cells (FCs). (B–I) Germaria with the control (B, C, F, G) or *brm^T362^* (D, E) or *bap180^Δ86^* (H, I) mutant GSC clones (broken circles) marked by the absence of GFP and the presence of an anteriorly anchored spectrosome (α-spectrin staining). In the wild type controls, marked GSCs are evident at 2 days and 14 days ACI (B, C, F, G). Conversely, marked GSCs mutant for *brm* (D) or *bap180* (H) are only detected at 2 days ACI, but lost at 14 days ACI (E, I). Instead, the mutant cyst clones are present in the germaria (arrows in E and I). (J, K) The control (J) and *brm* knockdown (K) germarium stained for α-spectrin and Vasa. While two GSCs are present in the control germarium, the mutant one contains only one GSC. GSCs are indicated by arrows. (L) Graph showing the relative percentage of germaria containing marked wild type control or *brm* or *bap180* mutant GSCs over a 3-week period ACI. Note that all initial percentages at day 2 ACI are normalized to 100%. (M) Graph showing that a gradual GSC loss is elicited by knocking down either *brm* or *bap180* in the germline.

Recent studies have shown that the BAP complex, one of the *Drosophila* SWI/SNF chromatin-remodeling complexes, regulates stem cell lineage commitment and stem cell proliferation in the adult *Drosophila* intestine [45], [46]. These findings prompted us to investigate if the *Drosophila* SWI/SNF complexes function in regulating the GSC fate in the ovary. For this purpose, we first examined whether Brm, the ATPase subunit of the *Drosophila* SWI/SNF complexes, has a role in GSC maintenance. The immunofluorescence assay using anti-Brm serum revealed that *brm* is ubiquitously expressed in almost all cell types in the wild type germaria including GSCs, the niche and follicle cells (Figure 1A and A′). To determine if loss-of-function mutations in *brm* perturb GSC self-renewal, we performed a clonal analysis in which GSC clones homozygous for either the mutant allele of *brm* or wild type control are generated by FLP/FRT-based mitotic recombination. The GSC clones can be identified by loss of GFP expression and the presence of an anteriorly anchored spectrosome in the germaria (Figure 1B–D). Statistic analysis showed that in contrast to that of the controls, the rate of GSC clones homozygous for *brm^T362^*, a null mutant allele with embryonic lethality, declines rapidly in a 21-day time course after clonal induction (ACI) (Figure 1L), suggesting a cell-autonomous role of Brm in sustaining the GSC population. To validate this observation, we knocked down *brm* in the germline by expressing either *UAS-brm RNAi* (B35211 from Bloomington *Drosophila* Stock Center, BDSC) or *UAS-brm^K804R^*, a dominant negative form of *brm* transgene under the control of *nos-Gal4* driver. As depicted in figure 1M, reduced expression of *brm* in the germline caused a marked decrease in GSC number per germarium during three weeks after fly eclosion. Combined with the clonal analysis above, the Gal4/UAS binary system-based assay indicates that Brm is required intrinsically for maintaining GSCs.

It is established that Brm functions in the form of either BAP or PBAP complex. We then sought to characterize the Brm-containing SWI/SNF complex in the context of GSC maintenance. To define the complex subtype functioning in the ovary, we first tested Osa, the BAP complex-specific subunit, for a potential role in maintaining GSCs. As adult escapers of female flies transheterozygous for *osa^2^*/*osa^308^* can be collected for an assay, we examined if loss of *osa* function impairs GSC self-renewal by directly counting GSC number at different time points after fly eclosion. Clearly, *osa^2^*/*osa^308^* females had a relatively constant number of GSCs per germarium with a 14 day test period, similar to the wild type counterparts (Table 1). Thus, these results do not support a possibility that Brm in the form of the BAP complex is involved in sustaining the GSC population. Polybromo/Bap180 is a signature subunit of the PBAP complex. To test the alternative that the PBAP, rather than BAP complex is required for GSC maintenance, we then analyzed how *polybromo* mutant GSCs are maintained using the clonal analysis described above (Figure 1F–I). As shown in figure 1L, the rate of GSC clones mutant for *bap180^Δ86^*, a null allele of *polybromo*, remarkedly decreased in a 21 day period ACI. Similarly, a gradual GSC loss was observed in a RNAi-based knock down experiment (RNAi transgenic line B32840 from the BDSC) (Figure 1M). All data above indicate that *polybromo* in the germline is essential for maintaining GSCs. To functionally link Brm to Polybromo, we further tested for possible genetic interactions of *brm* with *bap180* in controlling GSC self-renewal. In these experiments (Table 2), *brm^2^*/*+* or *bap180^Δ86^*/+ single heterozygous flies had a relatively constant number of GSCs per germarium during a 14 day test period, but *brm^2^*/*bap180^Δ86^* female flies that are double heterozygotes for both *brm* and *bap180* displayed a defect in sustaining the GSC population. In parallel, GSC maintenance within the test period remained normal in double heterozygotes for both *brm* and *osa* (Table 2). Thus, these studies identified a genetic interaction of *brm* with *bap180*, suggesting that the germline Brm functions in the form of the PBAP complex during GSC maintenance.

**Table 1:** *osa* mutations do not disrupt GSC maintenance in the ovary.

Phenotype	GSC number per germarium		
	2 day	7 day	14 day
*CS/w^1118^*	2.45 (n = 110)	2.45 (n = 104)	2.44 (n = 104)
*osa^2^/osa^308^*	2.51 (n = 109)	2.49 (n = 105)	2.45 (n = 96)

**Table 2:** *brm* genetically interacts with *bap180* but not *osa* in maintaining GSCs.

Phenotype	GSC number per germarium		
	2 day	7 day	14 day
*brm^2^/+*	2.33 (n = 108)	2.27 (n = 85)	2.11 (n = 83)
*bap180^Δ86^/+*	2.52 (n = 108)	2.36 (n = 105)	2.5 (n = 88)
*osa^2^/+*	2.45 (n = 106)	2.35 (n = 89)	2.31 (n = 87)
*brm^2^/bap180^Δ86^*	2.20 (n = 87)	1.39 (n = 82)*	1.45 (n = 84)*
*brm^2^/osa^2^*	2.47 (n = 108)	2.21 (n = 83)	2.21 (n = 82)

*p<0.05.

GSC maintenance defects elicited by the PBAP complex deficiency could be attributable to promoted cell death or precocious differentiation. To differentiate these possibilities, we performed an immunostaining assay using anti-Cleaved-Caspase 3 antibody. The experiment failed to detect any apoptotic signals in the marked *brm* mutant GSCs (data not shown). Given that the lost *brm* mutant GSCs are able to develop into differentiated germline cysts (Figure 1E), we argue that *brm* mutation-induced GSC loss might result from increased stem cell differentiation. BMP signaling plays a pivotal role in controlling GSC self-renewal through repressing differentiation via modulating transcriptional silencing of *bam*, a GSC differentiation promoting gene. To understand mechanisms underlying the defective GSC maintenance, we examined BMP signaling activities, as well as *bam* expression pattern in *brm* mutant GSC clones. The indirect immunofluorescence analyses revealed that while the mutant GSCs respond to BMP signals at the same magnitude as the neighboring wild type controls, repression of *bam* expression remains in the mutant GSCs (Figure S1A, B and data not shown). These data suggest that the cell-autonomous role of the PBAP complex in GSC maintenance is independent of BMP/Bam pathway.

### PBAP complex in the niche has a non-cell autonomous role in maintaining GSCs

**Figure 2:**
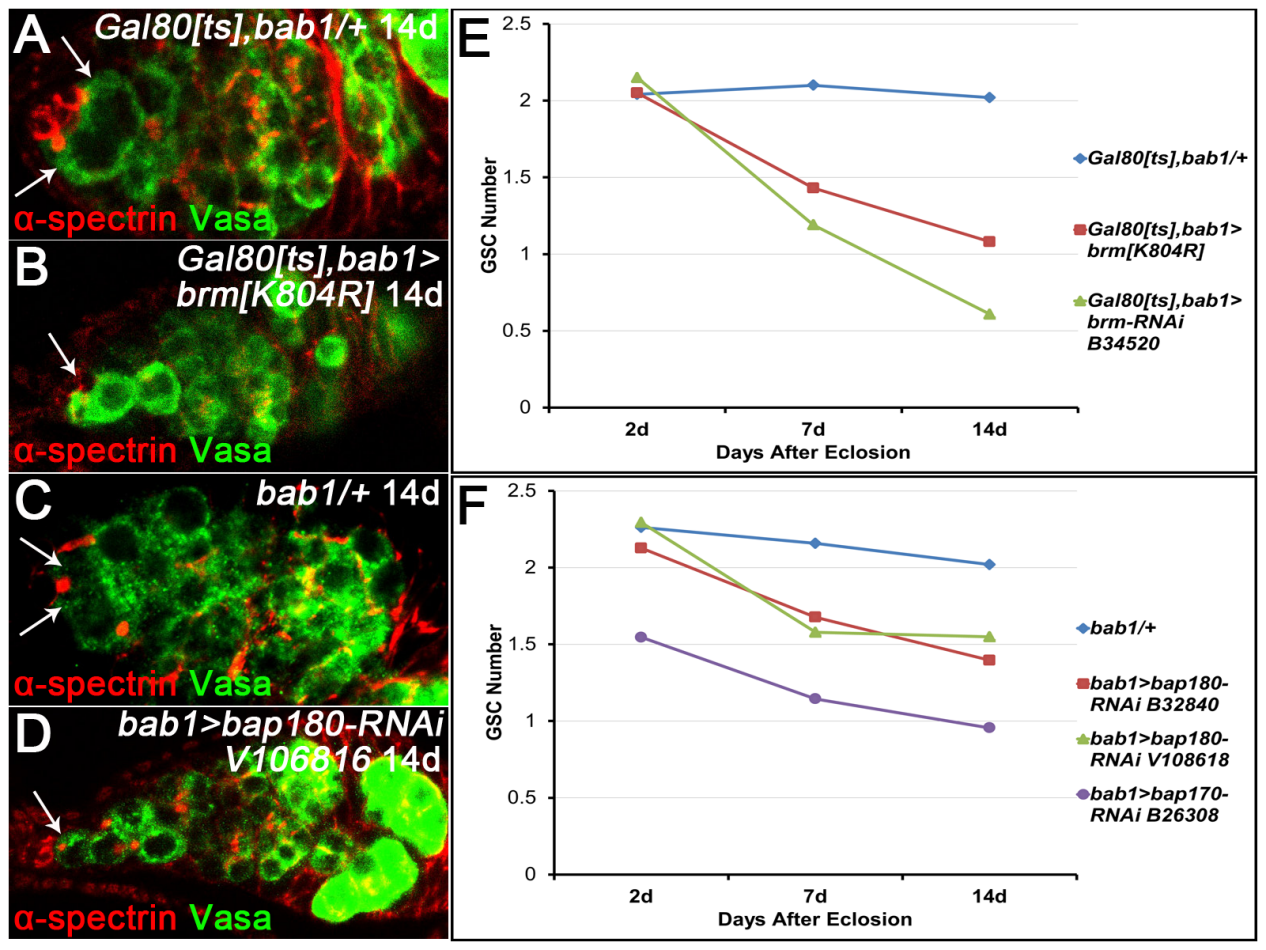
Knock down of the PBAP complex subunits in the niche leads to a gradual GSC loss. (A–D) The control germaria (A, C) and mutant ones expressing *brm-Dominant-Negative* (*brm[K804R]*) (B) or *bap180-RNAi* transgene (D) under the control of *bab1-gal4*, stained for Vasa and α-spectrin. Only one GSC is present in the knockdown germarium at 14 days after eclosion (B, D), whereas the control germarium contains two GSCs (A, C). GSCs are indicated by arrows in all panels. (E, F) Graphs show that compared with the controls, knocking down *brm* (E) or the PBAP specific subunit encoding gene (*bap180* or *bap170*) (F) in the niche causes a significant drop of GSC number per germarium over a 2-week period after eclosion.

Given that epigenetic regulation of GSC maintenance involves both intrinsic and extrinsic mechanisms [31], [32] and Brm expression is evident in the GSC niche (Figure 1A and A′), we assumed that the PBAP complex has also a non-cell autonomous role in controlling GSC self-renewal. To discern the possibility, we first combined *tubP-Gal80^ts^* with *UAS-brm RNAi* (B34520 from the BDSC) or *UAS-brm^K804R^* and *bab1-Gal4*, a GSC niche-specific driver, and performed a temperature shift assay. In the experiments, the females were raised at 18°C until 2 days after eclosion and then shifted to 29°C for a number of days. Cell number counting at different time points revealed that reduced expression of *brm* in the niche cells only at adulthood causes a gradual GSC loss (Figure 2A, B and E). Thus, this spatial-temporally controlled study supports the notion that Brm is required extrinsically for maintaining GSCs.

To verify if Brm in the niche acts in the form of the PBAP complex to control GSC self-renewal, we next tested polybromo and bap170, another specific subunit of the PBAP complex, for the non-cell autonomous roles in sustaining the GSC population. As depicted in figure 2C, D and F, RNAi-based knock down of either *polybromo* (B32840 from the BDSC, V108618 from Vienna *Drosophila* RNAi Center, VDRC) or *bap170* (B26308 from the BDSC) in the niche cells elicited a significant decrease in GSC number per germarium in a 14 day time course, albeit reduced GSC number at eclosion was observed in the case of *bap170* knock down. These data not only indicate extrinsic roles of *polybromo* and *bap170* in GSC maintenance, but also provide more evidence that the PBAP complex controls GSC self-renewal at multiple levels.

CpCs in the niche produce BMP signals for keeping GSCs in an undifferentiated and self-renewing state. To determine whether knocking down the PBAP complex perturbs the niche signaling output, we expressed *UAS-brm RNAi* (B35211 from the BDSC) in the niche cells and then examined the expression levels of pMad in GSCs located in the knockdown germaria. As evident in supplementary figure S1C and S1D, pMad expression was remarkedly reduced in a significantly higher percentage of the mutant GSCs than that of the control ones (31.21%, n = 141 vs 9.29%, n = 140), indicative of a compromised BMP signaling emitted from the knock down niche. These results suggest that the niche-specific *brm* knockdown impairs BMP signaling output, presumably eliciting a defective GSC maintenance phenotype.

### The PBAP complex is dispensable for germline differentiation within the germarium

**Figure 3:**
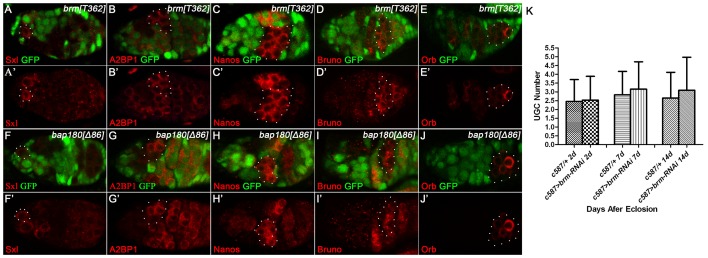
Removal or knock down of *brm* or *bap180* function does not disrupt germline differentiation within the germarium. (A–J′) Germaria containing *brm^T362^* (A–E′) or *bap180^Δ86^* (F–J′) mutant germ cell clones (broken circles) marked by the absence of nuclear GFP, stained for Sxl (A, A′, F, F′), A2BP1 (B, B′, G, G′), Nanos (C, C′, H, H′), Bruno (D, D′, I, I′) or Orb (E, E′, J, J′). GSC-derived germline differentiation within the germarium proceeds with dynamic expression of a number of molecular markers such as Sxl in GSCs/CBs (A, A′, F, F′), A2BP1 in germ cells starting from the 4-cell cysts (B, B′, G, G′), Nanos in 16-cell germline cysts (C, C′, H, H′), Bruno in germ cells of the 16-cell cysts (D, D′, I, I′) and Orb in oocyte of the 16-cell cysts (E, E′, J, J′). The expression pattern of all tested differentiation markers remains unchanged in the germline clones homozygous for either *brm^T362^* (A–E′) or *bap180^Δ86^* (F–J′). (K) Graph shows that compared with the controls, *brm* knockdown in ECs does not cause the accumulation of UGCs in the germarium over a 14-day time course after eclosion.

It has been shown that germline lineage commitment in the ovary proceeds with stepwise GSC-derived germ cell differentiation involving germline cyst development within the germarium, and epigenetic regulation is implicated in this process [30], [32]. In order to clarify whether the PBAP complex acts in the germline differentiation, we first generated germline clones homozygous for the *brm^T362^* allele using the FRT/FLP technique and analyzed how differentiation of GSCs and their derivatives occurs in *brm* mutant germaria at both molecular and morphological levels. As shown in figure 3A–E′, the expression pattern of all tested molecular markers for ranging from the early to late differentiation remained unchanged (Sex lethal, 100%, n = 23; A2BP1, 100%, n = 27; Nanos, 100%, n = 19; Bruno, 100%, n = 29; Orb, 95.65%, n = 23). In addition, we observed that the mature egg chambers in which the entire germ line is mutated for *brm* appear normal with respective to the cyst development (Figure 1E). Similarly, loss-of-function mutations in the germline *bap180* did not cause any molecular and morphological abnormality in the differentiation (Figure 1I, 3F–J′ and data not shown). Taken together, these studies indicate that the PBAP complex is not required cell-autonomously for controlling germline lineage commitment within the germarium.

Escort cells (ECs) in the germarium have recently been defined as the germline stem cell differentiation niche. Considering that *brm* is predominantly expressed in ECs, we next sought to exclude a possibility that the PBAP complex has a non-cell autonomous role in the germline differentiation. For this purpose, *UAS-brm-RNAi* transgene (B35211 from BDSC) was expressed under the control of *c587-Gal4*, restricting *brm* down-regulation to ECs and early follicle cells in adults. Same as the control group, reduced expression of *brm* in the somatic cells did not block GSC differentiation, as indicated by the absence of accumulation of the undifferentiated germ cells (UGCs) (Figure 3K). Thus, these data provide more evidence that the PBAP complex is dispensable for germline lineage differentiation within the germarium.

Previous studies have shown that the core subunits of the *Drosophila* SWI/SNF complexes, Brm, Mor and Snr1, are required in the germline for oogenesis [38], [47], [48], [49]. However, it remains to be determined how the Brm-containing complex functions in this process. In this paper, we identified a regulatory role for Brm in maintaining GSCs, while excluding its involvement in germline differentiation within the germarium. Moreover, our studies revealed that mutations in *polybromo/bap180*, rather than *osa*, cause a similar phenotype to that in *brm*, and *brm* genetically interacts with *bap180* in controlling GSC self-renewal. These genetic data lead us to propose that Brm acts in the form of the PBAP complex to sustain the GSC population. Given that requirements for the core Brm complex in oogenesis are independent of either BAP-specific subunit Osa or PBAP-specific subunits Bap180/Bap170 [38], we provide here the first demonstration that Brm functions in concert with Polybromo/Bap180 in early oogenesis, defining the *Drosophila* SWI/SNF complex subtype in the specific developmental context.

Like other epigenetic factors [31], [32], the PBAP complex controls GSC self-renewal both intrinsically and extrinsically. In the case of cell-autonomous manner, GSCs lacking Brm activity are able to respond properly to BMP signals emitted from the niche, sustaining the repression of the *bam* expression. This observation rules out the possibility that the canonical BMP/Bam pathway is implicated in Brm-controlled GSC maintenance. On the contrary, knocking down *brm* in the niche cells impairs BMP signaling output, probably eliciting a non-cell autonomous defect in GSC maintenance. Emerging evidence indicates that the chromatin-remodeling complexes execute unique developmental roles through interacting with a variety of transcription factors and/or other co-factors such as histone-modifying enzymes in a cell-type specific or developmental-stage specific manner [50]. Therefore, identification and functional characterization of the PBAP complex-binding partners, as well as epigenetically regulated target genes in the ovary would be beneficial for elucidating the above distinct mechanisms underlying intrinsic and extrinsic function of the PBAP complex in controlling GSC self-renewal. Given that the evolutionarily conserved SWI/SNF complexes have crucial roles in regulating both embryonic and adult stem cell fate in invertebrates, vertebrates and plants [24], [45], [50], mechanistic studies on PBAP complex-controlled GSC maintenance may shed lights on stem cell regulation in higher organisms.

## Supporting Information

Figure S1 ***brm*** **knock down in the niche, rather than loss of** ***brm*** **function in GSCs perturbs BMP signaling.** (A–D) Germaria with the control (A) or *brm^T362^* homozygous (B) GSC clone (broken circles) labeled by the absence of the nuclear GFP, or expressing *bab1-gal4* alone (C) or *brm-RNAi* with *bab1-gal4* (D), stained for pMad (A, B) or pMad and α-spectrin (C, D). Clearly, high levels of pMad expression are evident in both marked wild type control and *brm* mutant GSC (broken circles in A and B). By contrast, pMad expression in GSCs is remarkably reduced in the *brm* knock down germarium (arrowhead in D), compared with that in the control (arrow in C).
(TIF)

Figure S2 **Molecular validation of on-targeting effects of the RNAi transgenic strains.** (A, B) RT-PCR analysis shows that *actin-gal4* or *hs-gal4* induced expression of the *UAS*-*brm-RNAi* (A) or *UAS*-*bap180-RNAi* (B) transgene in the 3^rd^ instar larvae leads to a reduction in the expression of endogenous *brm* (A) or *bap180* (B) at mRNA levels. The presented gels are representative of three independent experiments.
(TIF)
