# Supplementary figures and images for: Evidence for Chromatin-Remodeling Complex PBAP-Controlled Maintenance of the Drosophila Ovarian Germline Stem Cells
Source: PLoS One. 2014 Jul 28;9(7):e103473. doi: 10.1371/journal.pone.0103473 (PMC4113433; doi:10.1371/journal.pone.0103473)

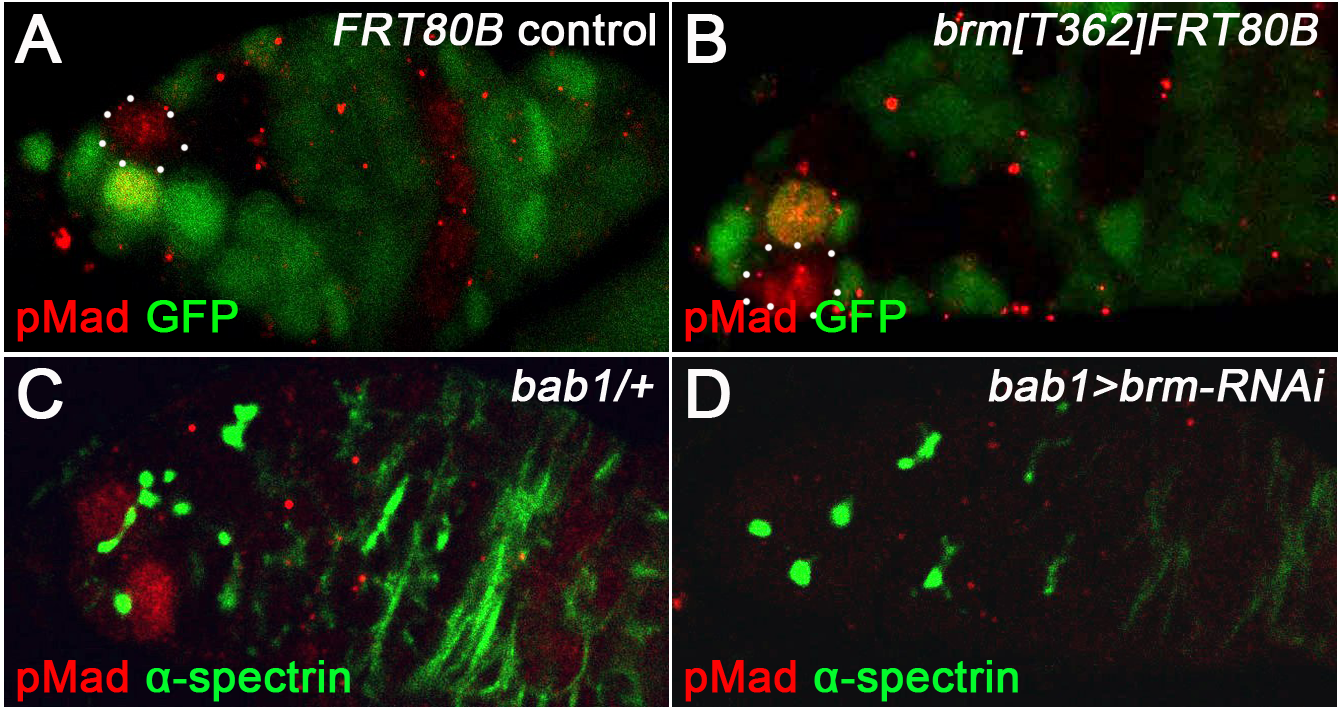

Supplement: Figure S1 — brm knock down in the niche, rather than loss of brm function in GSCs perturbs BMP signaling. (A–D) Germaria with the control (A) or brmT362 homozygous (B) GSC clone (broken circles) labeled by the absence of the nuclear GFP, or expressing bab1-gal4 alone (C) or brm-RNAi with bab1-gal4 (D), stained for pMad (A, B) or pMad and α-spectrin (C, D). Clearly, high levels of pMad expression are evident in both marked wild type control and brm mutant GSC (broken circles in A and B). By contrast, pMad expression in GSCs is remarkably reduced in the brm knock down germarium (arrowhead in D), compared with that in the control (arrow in C). (TIF) [file pone.0103473.s001.tif]

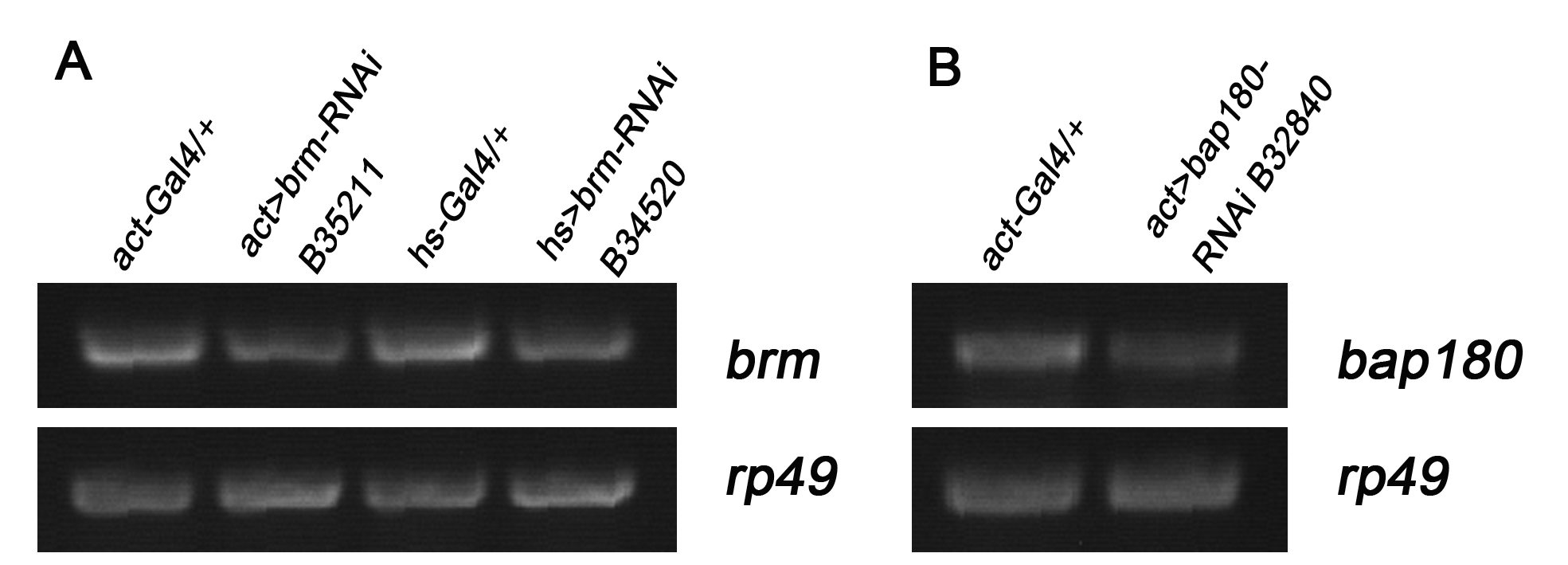

Supplement: Figure S2 — Molecular validation of on-targeting effects of the RNAi transgenic strains. (A, B) RT-PCR analysis shows that actin-gal4 or hs-gal4 induced expression of the UAS-brm-RNAi (A) or UAS-bap180-RNAi (B) transgene in the 3rd instar larvae leads to a reduction in the expression of endogenous brm (A) or bap180 (B) at mRNA levels. The presented gels are representative of three independent experiments. (TIF) [file pone.0103473.s002.tif]
